# Supplementary material for: Aggregation pathways of human γ D crystallin induced by metal ions revealed by time dependent methods
Source: PeerJ. 2020 Jun 12;8:e9178. doi: 10.7717/peerj.9178 (PMC7295030; doi:10.7717/peerj.9178)
Supplement: Supplemental Information 1 — Figure S1: Effect of Zn(II) and Cu(II) divalent metal ions as reported by turbidity assays. Figure S2: DLS measurement of HγD at 37 °C at different times. Figure S3: Effect of Zn and Cu divalent metal ions on the hydrodynamic radius. Figure S4: Different replicas of protein oligomerization induced by metal-binding. Figure S5: Fluorescence spectrum of HγD at diferents time in absense and presence of metals ion. Figure S6: Cu(II) binding to HγD crystallin as detected by NMR. Figure S7: HSQC spectra of HγD crystallin at different times. Figure S8: HγD conformational changes induced by metal ion followed by real time NMR. Figure S9: Intensity decay for selected residues followed by real time NMR. Figure S10: Scheme of unfolding region of HγD crystallins in presence of 1.5 equivalents of Cu (II) by NMR. Figure S11: Potentially binding sites of Zn (II) obtained by MIB and NMR. Figure S12: Potentially binding sites of Cu (II) obtained by MIB and NMR. [file peerj-08-9178-s001.pdf]

Aggregation pathways of human γ D crystallin induced by metal ions revealed by time dependent methods.

Arline Fernandez-Silva[1] , Leidys French-Pacheco[1,2], Lina Rivillas-Acevedo[2*], Carlos Amero[1*]

[1]Centro de Investigaciones Químicas, IICBA, Universidad Autónoma del Estado de Morelos, Cuernavaca, Morelos, Mexico.
[2]Centro de Investigación en Dinámica Celular, IICBA, Universidad Autónoma del Estado de Morelos, Cuernavaca, Morelos, Mexico.

Corresponding Author:
Carlos Amero, Centro de Investigaciones Químicas, IICBA, Universidad Autónoma del Estado de Morelos, Cuernavaca, Morelos, Mexico. carlosamero@uaem.mx

Lina Rivillas-Acevedo, Centro de Investigación en Dinámica Celular, IICBA, Universidad Autónoma del Estado de Morelos, Cuernavaca, Morelos, Mexico. lrivillas@uaem.mx

Abstract
Cataract formation is a slow accumulative process due to protein aggregates promoted by different factors over time. Zinc and copper ions have been reported to induce the formation of aggregates opaque to light in the human gamma D crystallin (HγD) in a concentration and temperature dependent manner. In order to gain insight into the mechanism of metal-induced aggregation of HγD under conditions that mimic more closely the slow, accumulative process of the disease, we have studied the non-equilibrium process with the minimal metal dose that triggers HγD aggregation. Using a wide variety of biophysics techniques such as turbidimetry, dynamic light scattering, fluorescence, nuclear magnetic resonance and computational methods, we obtained information on the molecular mechanisms for the formation of aggregates. Zn(II) ions bind to different regions at the protein, probably with similar affinities. This binding induces a small conformational rearrangement within and between domains and aggregates via the formation of metal bridges without any detectable unfolded intermediates. In contrast, Cu(II)-induced aggregation includes a lag time, in which the N-terminal domain partially unfolds while the C-terminal domain and parts of the N-terminal domain remain in a native-like conformation. This partially unfolded intermediate is prone to form the high-molecular weight aggregates. Our results clearly show that different external factors can promote protein aggregation following different pathways.

SUPPLEMENTARY FIGURES:

Figure S1: Effect of Zn(II) and Cu(II) as reported by turbidity assays at 37 °C for 12 h.

Figure S2: DLS measurement at 37 °C and different times.

Figure S3: Effect of Zn(II) and Cu(II) on the hydrodynamic radius (Z average) of the sample at 37 °C for 12 h.

Figure S4: Different replicas of protein oligomerization induced by metal-binding.

Figure S5: Normalized fluorescence spectra at different times.

Figure S6: Cu(II) binding to HγD crystallin as detected by NMR.

Figure S7: ^{1}H ^{15}N-HSQC spectra of HγD crystallin at different times.

Figure S8: HγD conformational changes induced by Zn(II) followed by real time NMR.

Figure S9: **I**ntensity decay for selected HγD residues in the presence of 1.5 equivalent of Cu(II) followed by real time NMR.

Figure S10: Scheme of unfolding region of HγD crystallins in presence of 1.5 equivalents of Cu(II) by NMR.

Figure S11: Potentially binding sites of Zn(II) predicted by MIB and NMR.

Figure S12: Potentially binding sites of Cu(II) obtained by MIB and NMR.

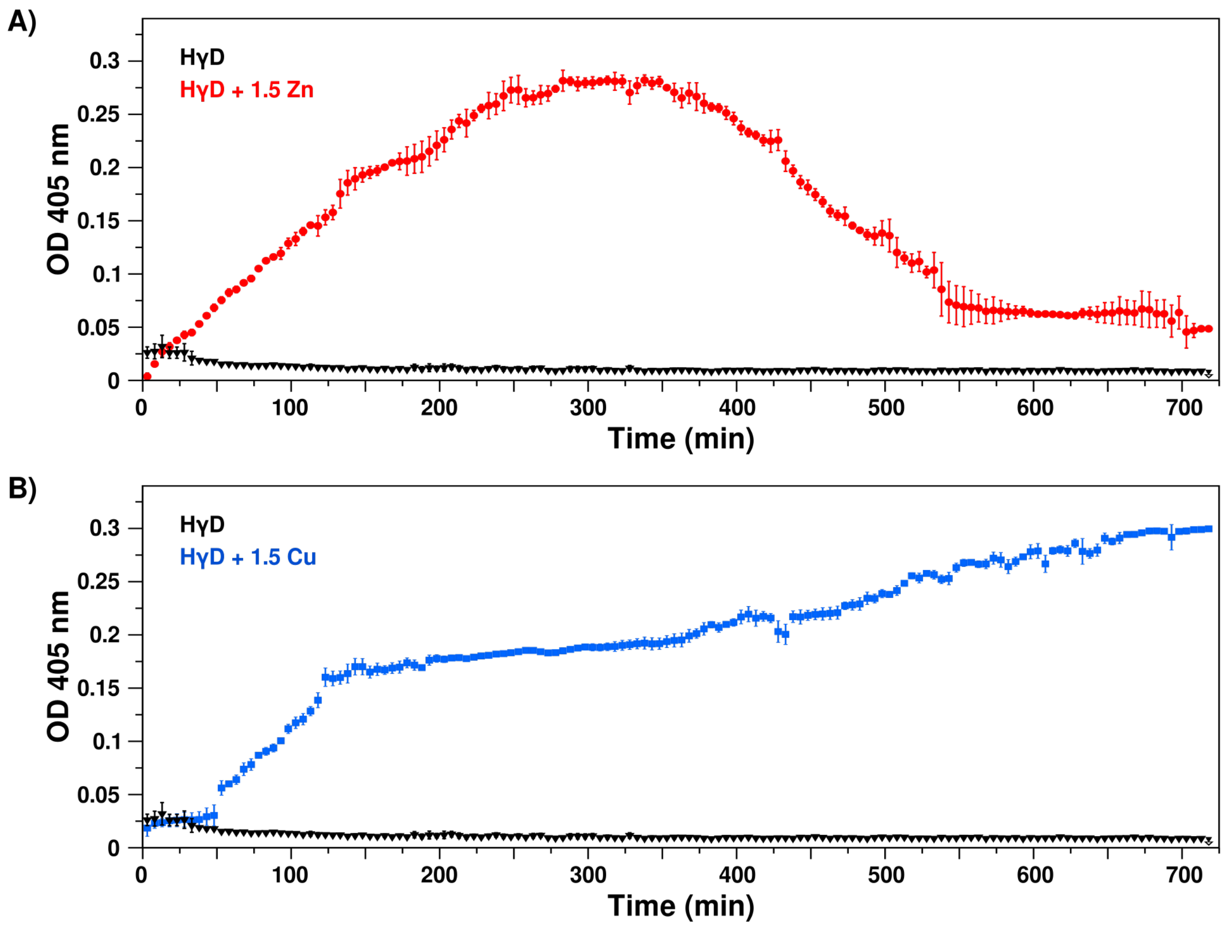

Figure S1: Effect of Zn(II) and Cu(II) as reported by turbidity assays at 37 °C for 12 h.

A) Absorbance at 405 nm as function of time of HγD crystallin in the absence (black line) and presence of 1.5 equivalents of Zn(II) (red).

B) HγD crystallin in the absence (black line) and presence of 1.5 equivalents of Cu(II) (blue). The observed decrease in turbidity at longer times correspond to the appearance of precipitation in the cuvette.

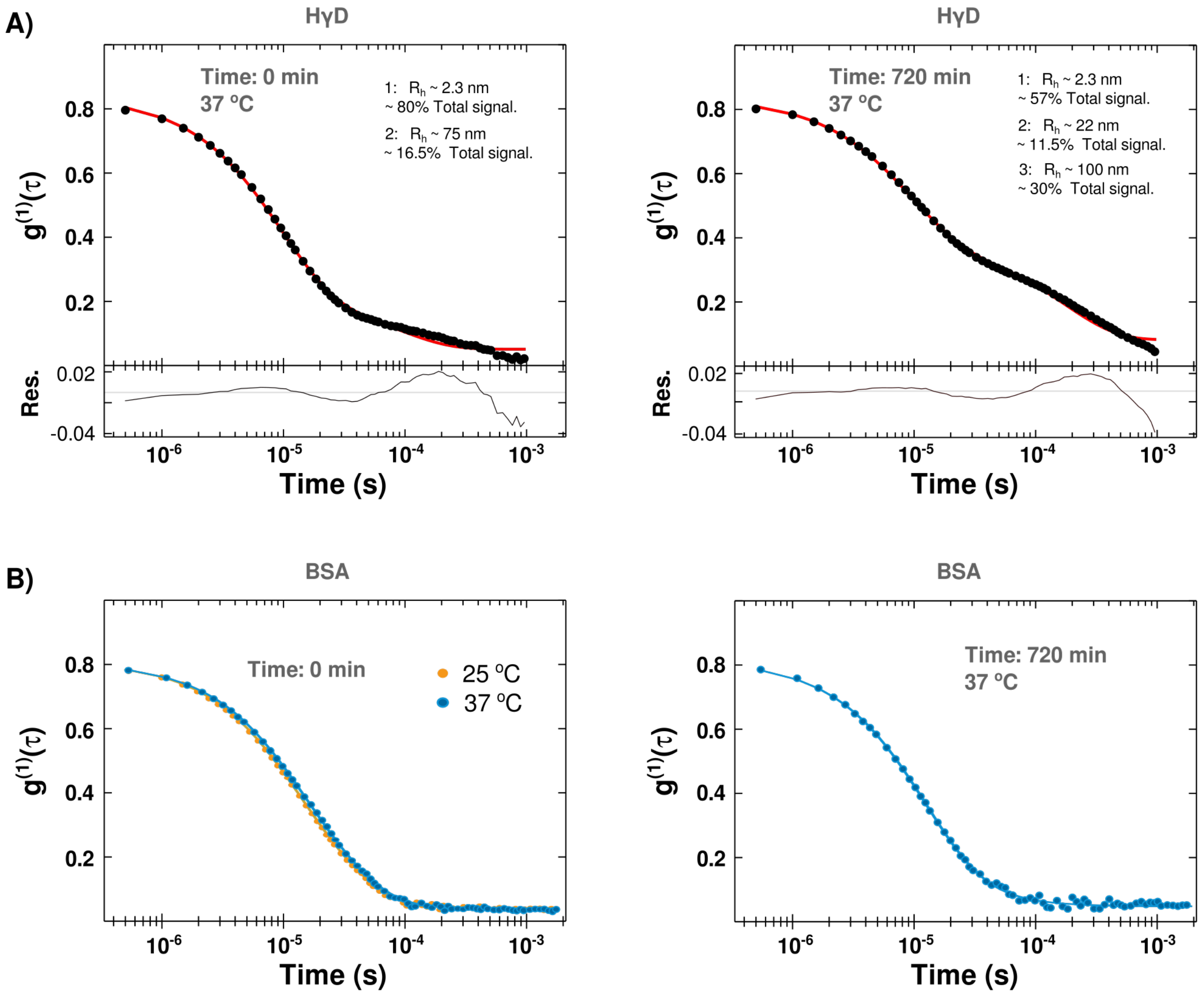

Figure S2: DLS measurement at 37 °C and different times.

A) DLS measurement of HγD at 37 °C at time 0 (right), shows a polydisperse curve which was not well described by a single population. The best fitting (red line) was obtained by a distribution with two different populations. Data of HγD after 12 h (left).

B) DLS measurement of BSA at 25 and 37°C as control with the same conditions (right). The data shows a monodisperse curve which was described by a single population. Data of BSA after 12 h at 37 °C (left)

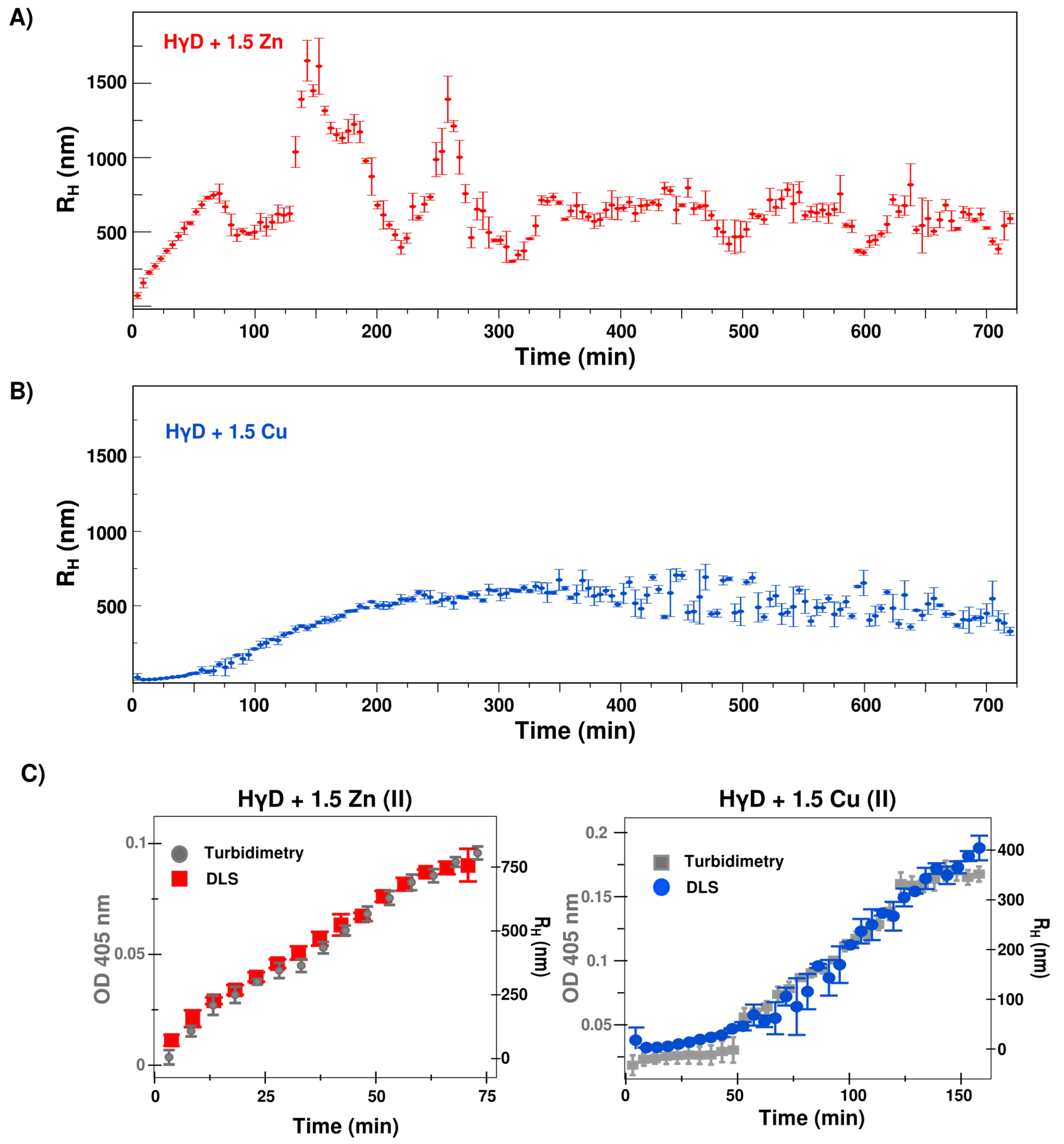

Figure S3: Effect of Zn(II) and Cu(II) on the hydrodynamic radius (Z average) of the sample at 37 °C for 12 h.

A) Estimated radius as function of time of HγD crystallin in the presence of 1.5 equivalents of Zn(II). B) Estimated radius as function of time of HγD crystallin in the presence of 1.5 equivalents of Cu(II). C) Overlay of the changes in turbidimetry (grey) and the estimated hydrodynamic radius as a function of time in the presence of Zn(II) (left) and in the presence of Cu(II) (right).

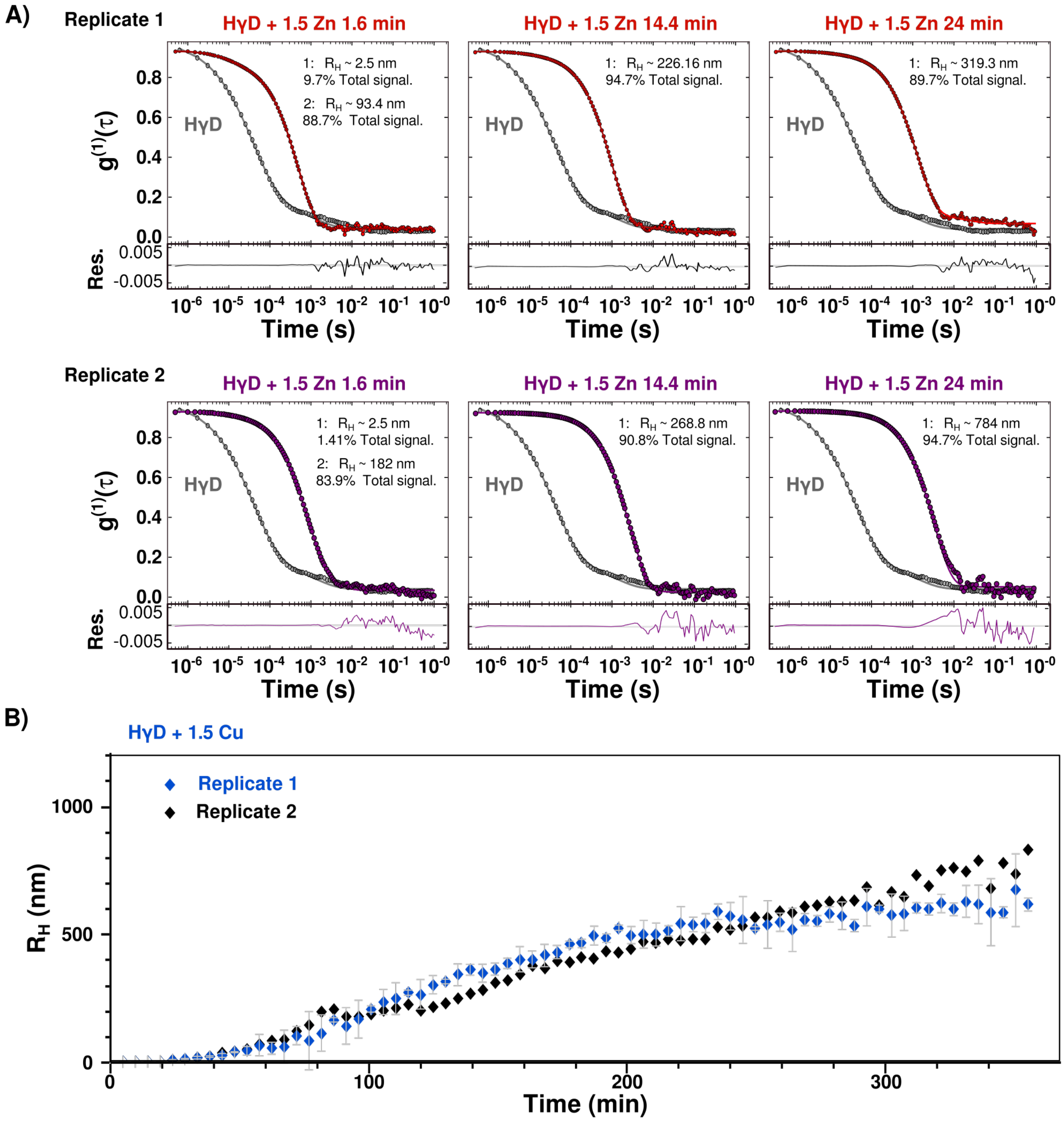

Figure S4: Different replicas of protein oligomerization induced by metal-binding.

A) Correlation coefficient of HγD replica 1 in the absence (grey line) and presence of 1.5 equivalents of Zn(II) (red) at 1.6 min, 14.4 min and 24 min after the addition of the metal ion.
B) Correlation coefficient of HγD replica 2 in the absence (grey line) and presence of 1.5 equivalents of Zn(II) (purple) at 1.6 min, 14.4 min and 24 min after the addition of the metal ion.
C) Effect of Cu(II) on the HγD hydrodynamic radius (Z average) of the sample at 37 °C for 12 h. Replica 1 is shown in black. Replica 2 is show in blue.

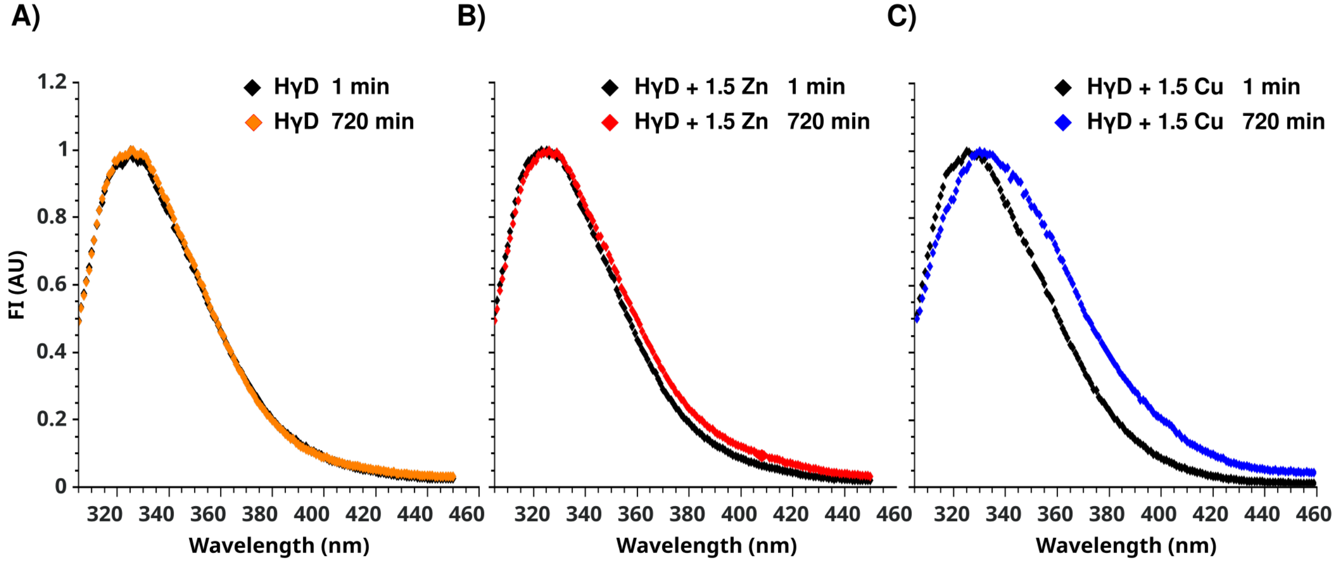

Figure S5: Normalized fluorescence spectra at different times.

A) HγD in the absence of metal ions, minute 1 (black) and 720 min (orange). B) HγD in the presence of Zn(II), minute 1 (black) and 720 min (red). C) HγD in the presence of Cu(II), minute 1 (black) and 720 min (red).The emission spectrum was recorded in the range of 300 and 500 nm using an excitation wavelength of 295 nm at 37 °C.

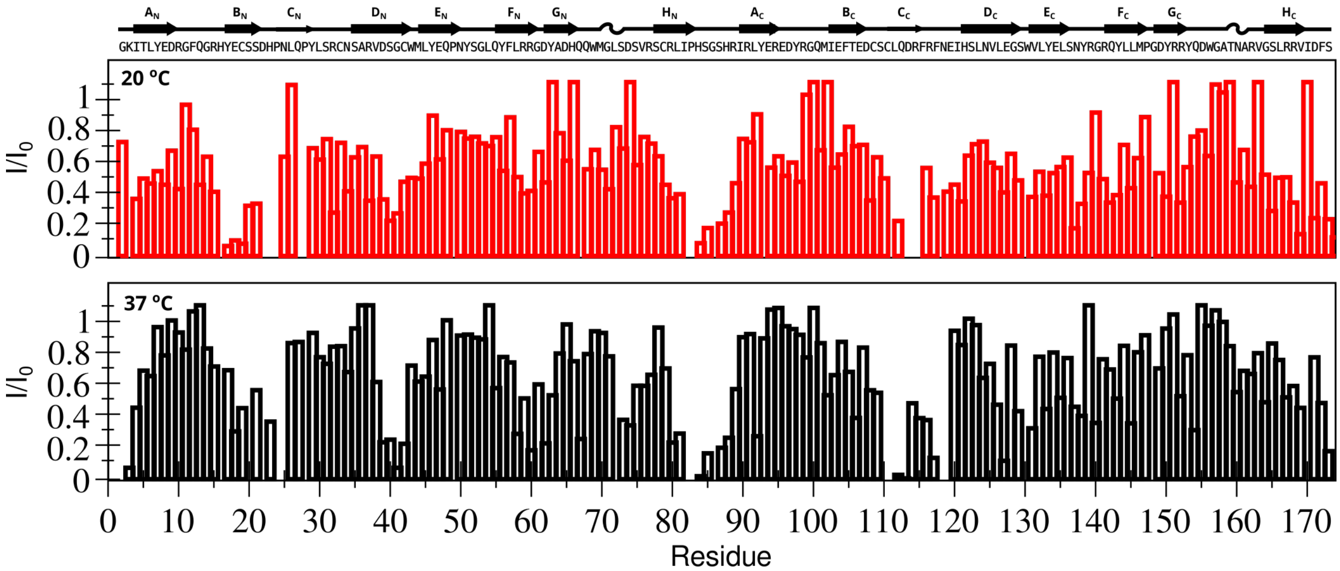

Figure S6: Cu(II) binding to HγD crystallin as detected by NMR.

Weighted intensity change (I/Io profiles) signals of HγD after the addition of Cu(II) at 20 °C (top) and 37 °C (bottom).

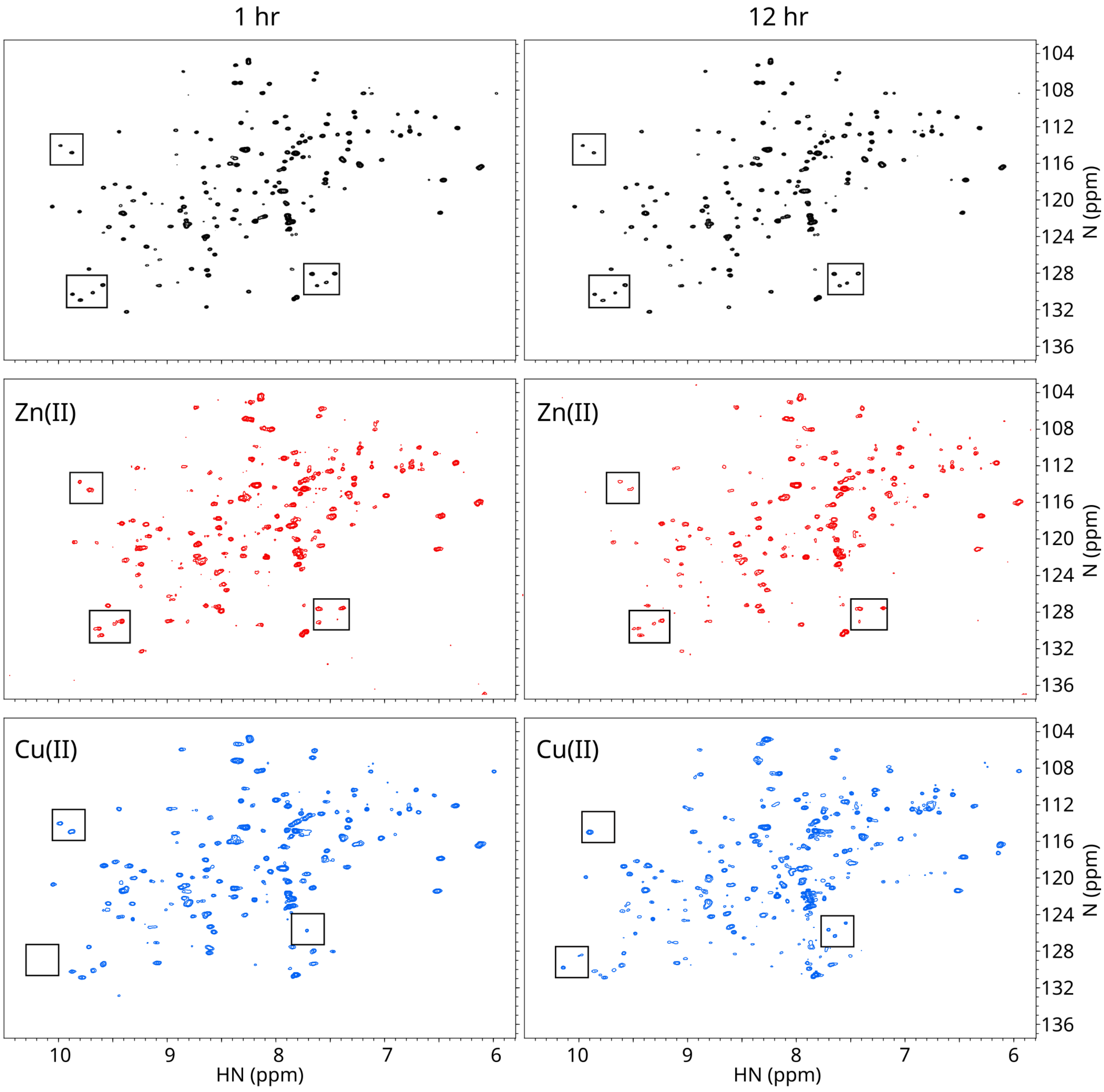

Figure S7: ^{1}H ^{15}N-HSQC spectra of HγD crystallin at different times.

Spectra in the absence (black) and presence of 1.5 equivalents of Zn(II) (red) and Cu(II) (blue). Residues in boxes are shown as examples of signals at one hour and 12 h.

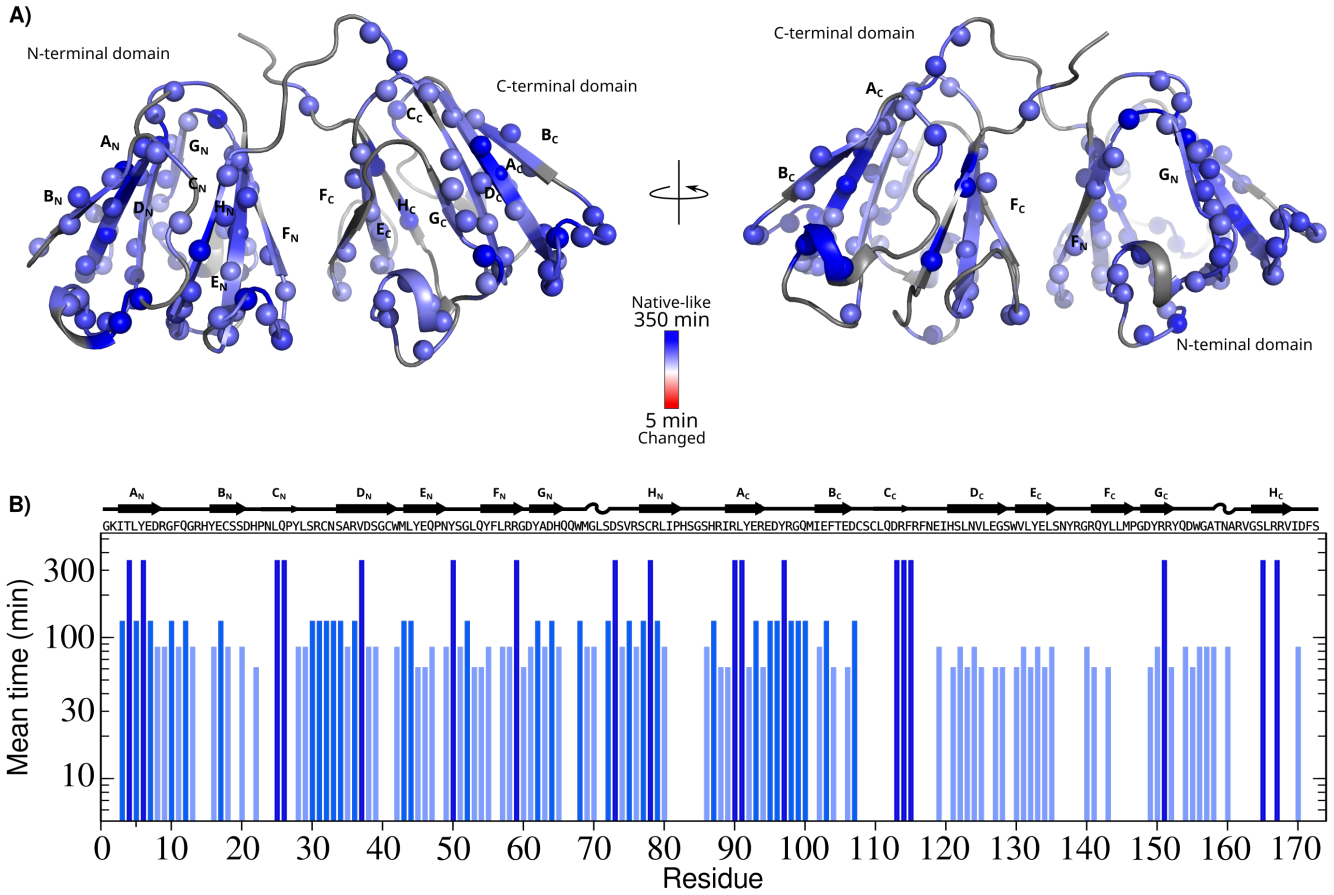

Figure S8: HγD conformational changes induced by Zn(II) followed by real time NMR.

A) Intensity mean time changes mapped onto the crystal structure of HγD crystallin (1HK0) and color-coded based on the profiles with a linear gradient ramp from dark blue (no changes) to red (small mean time). Unassigned residues are shown in dark gray.

B) Intensity mean-time changes (I/Io profiles) for the HSQC signals over time for each residue with Zn(II).

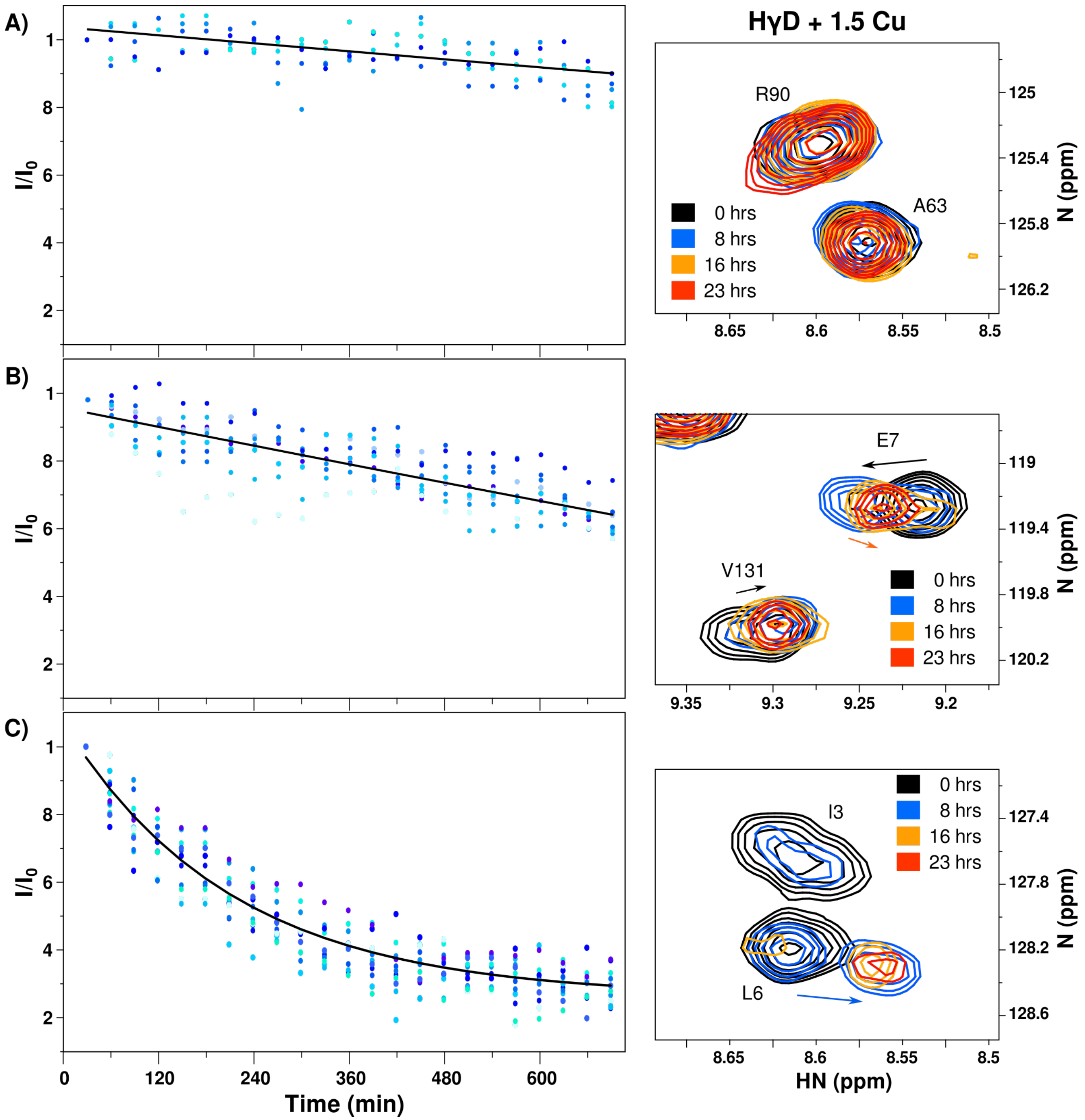

Figure S9: Intensity decay for selected HγD residues in the presence of 1.5 equivalent of Cu(II) followed by real time NMR.

A) Group 1: residues whose signal intensities or positions do not change substantially during the experiment. B) Group 2 and 3: residues with a small and medium decrease in their intensities. C) Group 4: residues where the intensity substantially decreases over time, and native-like resonance peaks almost disappear.

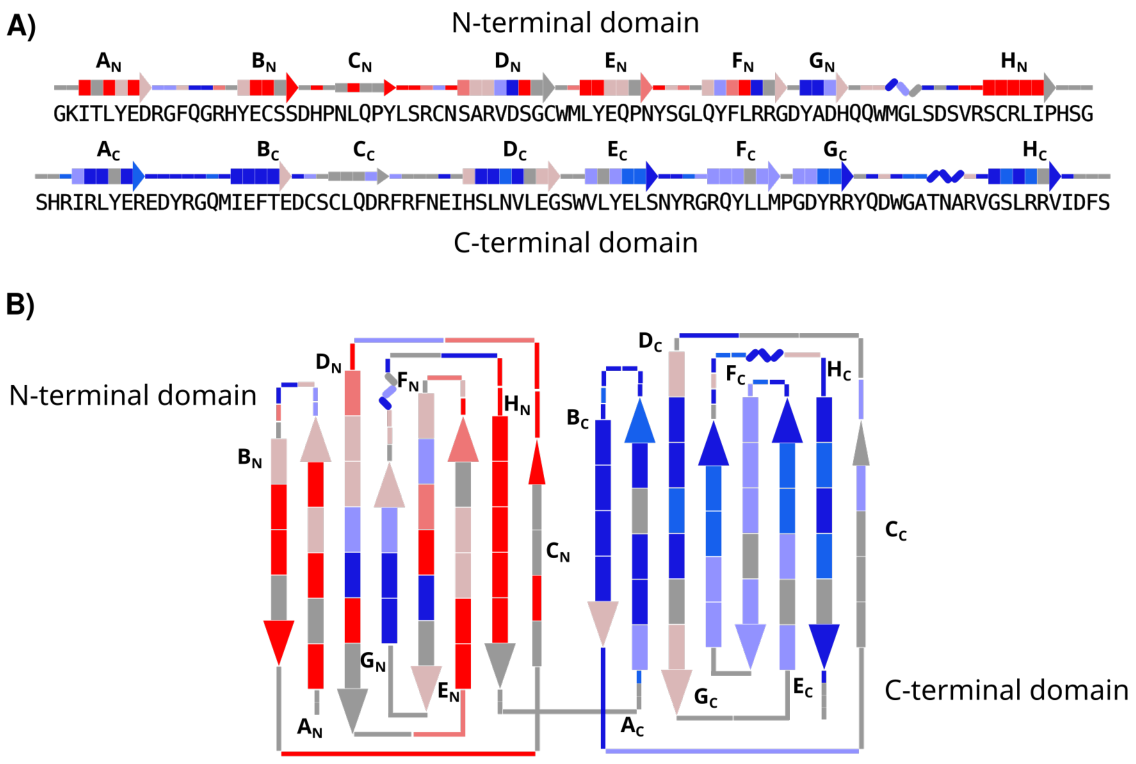

Figure S10: Scheme of unfolding region of HγD crystallins in presence of 1.5 equivalents of Cu(II) by NMR.

A) Secondary structure elements for both domain are shown with a color-coded based on the profiles with a linear gradient ramp from dark blue (no changes) to red (change). Unassigned residues are shown in gray. B) Same information described in topology of the N-terminal and C-terminal domain describing the four greek motifs.

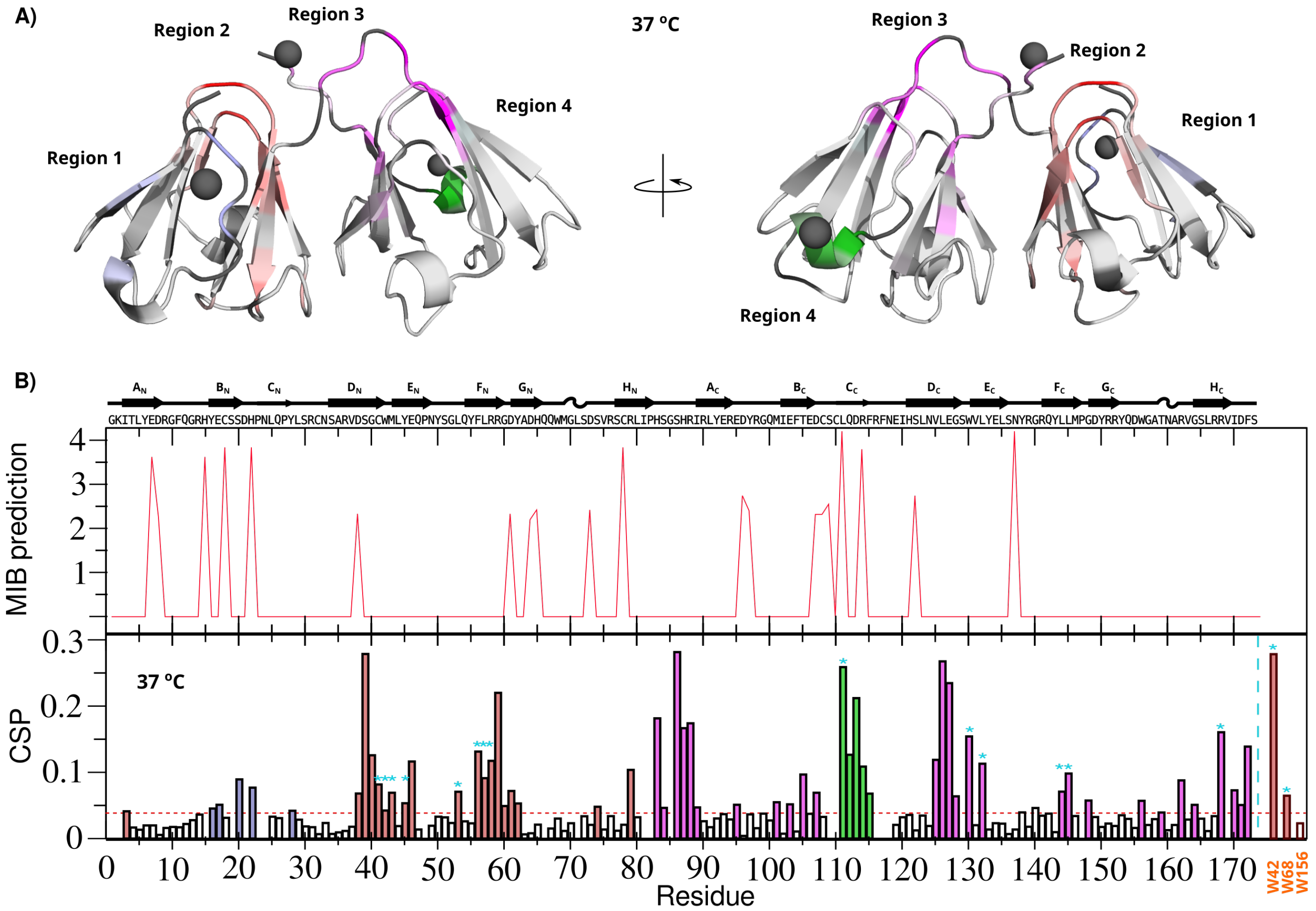

Figure S11: Potentially binding sites of Zn(II) predicted by MIB and NMR.

A) Amino acid side chains of four regions predicted from MIB and NMR as binding sites to Zn(II) at 37 °C mapped onto the protein structure (1HK0). Amino acids of region 1 (S20, D21, H22) (blue), region 2 (G40, C41, I81, H83) (red), region 3 (H87, E127) (purple), and region 4 (D113) (green).

B) Top: Zn(II) binding sites prediction of MIB software. Bottom: Chemical shift perturbation (CSP) of HγD after the addition of Zn(II) at 37 °C. Bars share the same color-code: region 1 (blue), region 2 (red), region 3 (purple) and region 4 (green). Cyan asterisks show buried residues that suffer significant changes. Side chain CSP for W42, W68 and W130 are shown at the far right.

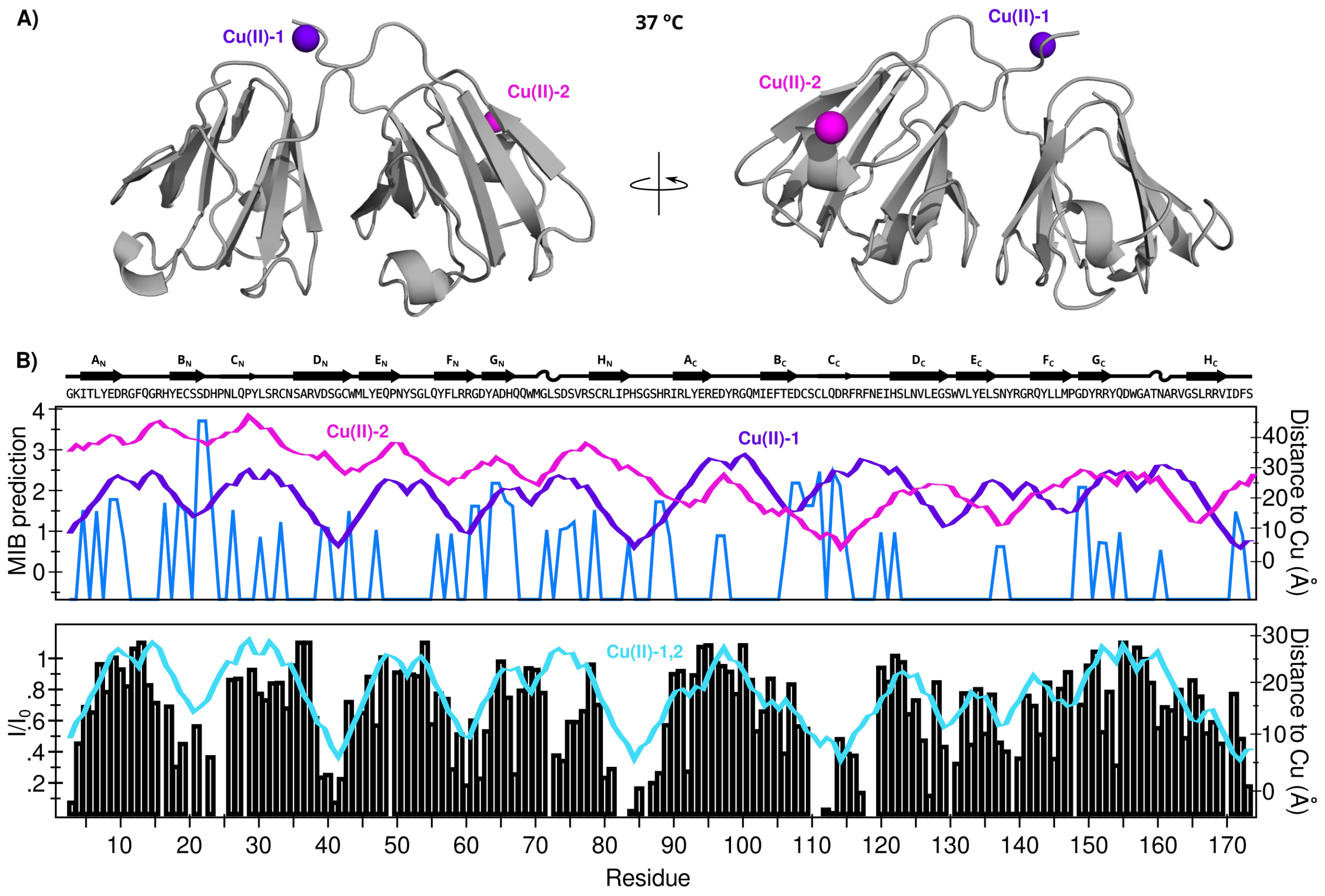

Figure S12: Potentially binding sites of Cu(II) obtained by MIB and NMR.

A) Amino acid side chains of four regions predicted from MIB and NMR as binding sites to Zn(II) at 37 °C mapped onto the protein structure (1HK0). Amino acids of region 1 (S20, D21, H22) (blue), region 2 (G40, C41, I81, H83) (red), region 3 (H87, E127) (purple), and region 4 (D113) (green).

B) Top: Cu(II) binding sites prediction from MIB software. Magenta line shows the distance between every HγD residue and copper at position Asp 113. Purple line shows the distance between every HγD residue and copper at His 83 - His 87.

Bottom: Overlaid weighted intensity change (I/Io profiles) signals of HγD after the addition of Cu(II) at 37 °C. Blue line shows the minimum distance between every HγD residue and either copper at position Asp 113 or His 83 - His 87.
